# Histopathology reveals correlative and unique phenotypes in a high-throughput mouse phenotyping screen

**DOI:** 10.1242/dmm.015263

**Published:** 2014-03-20

**Authors:** Hibret A. Adissu, Jeanne Estabel, David Sunter, Elizabeth Tuck, Yvette Hooks, Damian M. Carragher, Kay Clarke, Natasha A. Karp, Sanger Mouse Genetics Project, Susan Newbigging, Nora Jones, Lily Morikawa, Jacqueline K. White, Colin McKerlie

**Affiliations:** 1 Centre for Modeling Human Disease, Toronto Centre for Phenogenomics, 25 Orde Street, Toronto, ON M5T 3H7, Canada.; 2 Physiology and Experimental Medicine Research Program, The Hospital for Sick Children, 555 University Avenue, Toronto, ON M5G 1X8, Canada.; 3 Department of Laboratory Medicine and Pathobiology, Faculty of Medicine, University of Toronto, Toronto, ON M5S 1A8, Canada.; 4 Mouse Genetics Project, Wellcome Trust Sanger Institute, Wellcome Trust Genome Campus, Hinxton, Cambridge CB10 1SA, UK.

**Keywords:** Histopathology, High-throughput phenotyping, Mouse, Pathology

## Abstract

The Mouse Genetics Project (MGP) at the Wellcome Trust Sanger Institute aims to generate and phenotype over 800 genetically modified mouse lines over the next 5 years to gain a better understanding of mammalian gene function and provide an invaluable resource to the scientific community for follow-up studies. Phenotyping includes the generation of a standardized biobank of paraffin-embedded tissues for each mouse line, but histopathology is not routinely performed. In collaboration with the Pathology Core of the Centre for Modeling Human Disease (CMHD) we report the utility of histopathology in a high-throughput primary phenotyping screen. Histopathology was assessed in an unbiased selection of 50 mouse lines with (*n*=30) or without (*n*=20) clinical phenotypes detected by the standard MGP primary phenotyping screen. Our findings revealed that histopathology added correlating morphological data in 19 of 30 lines (63.3%) in which the primary screen detected a phenotype. In addition, seven of the 50 lines (14%) presented significant histopathology findings that were not associated with or predicted by the standard primary screen. Three of these seven lines had no clinical phenotype detected by the standard primary screen. Incidental and strain-associated background lesions were present in all mutant lines with good concordance to wild-type controls. These findings demonstrate the complementary and unique contribution of histopathology to high-throughput primary phenotyping of mutant mice.

## INTRODUCTION

The laboratory mouse is an invaluable model for functional annotation of mammalian genomes, and for improving our understanding of the genetic basis of normal human biology and disease (Brown et al., 2009). The International Knockout Mouse Consortium (IKMC) aims to mutate all protein-coding genes in the mouse using gene targeting and gene trapping in C57BL/6N embryonic stem (ES) cells (Skarnes et al., 2011; Bradley et al., 2012). Currently, the IKMC ES cell resource contains targeted alleles for over three-quarters of all protein-coding genes (www.mousephenotype.org), with complete genome coverage for protein-coding genes expected in a few years (Brown and Moore, 2012). The subsequent production and characterization of mice derived from this ES cell resource is undertaken by members of the International Mouse Phenotyping Consortium (IMPC) (Brown et al., 2009). Phase 1 of the IMPC program is funded to run from 2011 to 2016 to generate and phenotype 5000 mouse lines, with the challenge of a further 15,000 to be completed by 2021. Mutant lines undergo a standardized battery of clinical phenotyping tests encompassing a range of biomedical characteristics in order to assess the phenotypic consequence of the targeted allele, thereby generating biologically relevant, functional annotation of genes (Kim et al., 2010; Brown and Moore, 2012). The resulting biological resource and phenotyping data are openly available to the scientific community through web-based interfaces (Mallon et al., 2012).

The Sanger Institute Mouse Genetics Project (MGP) is one of the founding members of the IMPC and has, to date, generated over 1000 knockout first conditional ready mouse lines. The MGP phenotyping pipeline (White et al., 2013) aligns closely with the IMPC pipeline and includes more than 280 clinical parameters encompassing key biomedical areas such as reproduction, development, infection and immunity, musculoskeletal system, metabolism, and endocrinology. To date, this analysis has been completed and data released for over 720 lines of mice (http://www.sanger.ac.uk/mouseportal/). Summaries can be found by searching for each gene of interest in Wikipedia (http://en.wikipedia.org/wiki/Category:Genes_mutated_in_mice) and Mouse Genome Informatics (http://www.informatics.jax.org/). An important resource collected for every mutant mouse line as part of the standard MGP primary phenotyping screen is a tissue biobank, access to which can be requested by the community. This is generated from a comprehensive set of 42 organs and tissues that are collected at necropsy from 16-week-old mice, fixed for preservation, and processed to paraffin blocks. Currently, these paraffin blocks are archived and no histopathology is performed as part of the primary screen.

Pathology plays a pivotal role in bespoke, hypothesis-driven investigations of mouse models, providing important insight into the morphological consequences and mechanisms of gene function (Schofield et al., 2011). Ideally, high-throughput pathology screening would become an integral part of the IMPC primary phenotyping screen. However, labor cost and operational limitations (such as number of mice and the standardized set of tissues collected), combined with the paucity of established and standardized high-throughput pathology protocols, expertise in the spontaneous and induced pathology of genetically engineered mouse models, and data-capture and annotation tools to generate machine-searchable datasets is limiting its utility. As a result, the inclusion of pathology in a high-throughput phenotyping screen is dependent on the capacity of individual centers, and is often limited to mouse lines with a clear phenotype detected from the pipeline (Schofield et al., 2011). Furthermore, in many instances, histopathology analysis is restricted to anticipated target tissues and organs based on the focus of the research group, the existing knowledge base including unpublished phenotyping data such as identification of gross abnormalities, or gene expression data; hence, the majority of tissues and organs that are considered irrelevant are not analyzed. This non-comprehensive, ascertainment-biased approach indisputably carries the risk of missing important pathology findings and additional phenotypes, diminishes the IMPC’s objective to generate the most useful comprehensive and compelling phenotype descriptions, and undermines contextual pathophysiological explanations of disease mechanisms for the observed phenotype. This is particularly important in the context of gene pleiotropy whereby a single gene regulates more than one biological function (Beckers et al., 2009). For instance, many mutant lines analyzed by large-scale phenotyping initiatives show more than one phenotype annotation (Tang et al., 2010; Brown and Moore, 2012; White et al., 2013). Furthermore, a fully manifested phenotype is often a function of environmental (such as nutrition or microbiota) and intrinsic (such as age) factors (Beckers et al., 2009; Schofield et al., 2012). Histopathology could reveal early and subtle lesions before they are severe enough to cause detectable phenotypes, and, for some mutant lines, might represent the only detected abnormality (Schofield et al., 2012). To date, there has been no systematic study to evaluate the complementary and the unique contribution of histopathology in high-throughput mouse phenotyping.

**TRANSLATIONAL IMPACT**

**Clinical issue**

Light microscopic examination of tissue to evaluate the morphological manifestations of disease is an important and often essential part of clinical diagnosis. This approach, known as histopathology, also plays a pivotal role in hypothesis-driven biomedical research that uses mouse models of human disease. Histopathological evaluation of structural abnormalities in mutant mouse models provides important insights into gene function and mechanisms of pathogenesis, by identifying the morphological consequences of genetic mutations that are linked to disease. However, the scientific value, utility and feasibility of histopathology in large-scale hypothesis-generating clinical screening of mutant mice have not been systematically evaluated. Furthermore, histopathology is not routinely part of high-throughput phenotyping pipelines, including that devised by the International Mouse Phenotyping Consortium to generate and characterize a knockout line for every protein-coding gene in the mouse.

**Results**

In this study, the investigators conducted a detailed histopathology analysis of knockout mouse lines representing 50 protein-coding genes, to determine the utility of including histopathology in high-throughput phenotyping. Thirty of the lines had one or more clinical abnormality detected by a standard primary phenotyping screen. No clinical abnormalities were detected in the remaining 20 lines. Histopathology corroborated the clinical phenotypes observed in 19 of the 30 lines (63.3%) with clinical abnormalities, providing tissue and cellular morphology data that enhanced the description of the phenotype in each of these mutant mouse lines. Furthermore, in seven of the 50 lines (14%), histopathology revealed significant morphological abnormalities that were neither associated with nor predicted by clinical changes detected in the standard primary phenotyping screen. Three of these seven lines had no clinical abnormality based on the standard primary phenotyping screen.

**Implications and future directions**

This study demonstrates the value of including histopathology in high-throughput mouse phenotyping screens. Histopathology enhances the evaluation of knockout mice by providing corroborative data, but also by revealing phenotypes that are not readily detectable using other assays. Moreover, the approach provides a wealth of useful information on the tissue changes associated with mutant phenotypes, which can be directly relevant to human disease phenotypes. Finally, the authors demonstrate that histopathology can be integrated into phenotyping pipelines in a cost-effective way, supporting the inclusion of this technique in large-scale primary screens of targeted knockouts.

Because expanding the primary screen to include histopathology was predicted to complement and extend the MGP’s current interrogation of the phenotypic consequences of each targeted allele, we undertook a pilot screen in which histopathology was assessed using the biobanked samples from 50 mutant lines that had completed the MGP primary screen. Phenodeviant characteristics had been identified and annotated for 30 of these lines, whereas the primary screen had not detected any abnormality in the remaining 20 lines selected for this pilot. Here, we report the value of including comprehensive, systematic histopathology as part of a high-throughput primary phenotyping screen.

## RESULTS

### Findings from the MGP primary phenotyping screen

**Fig. 1:**
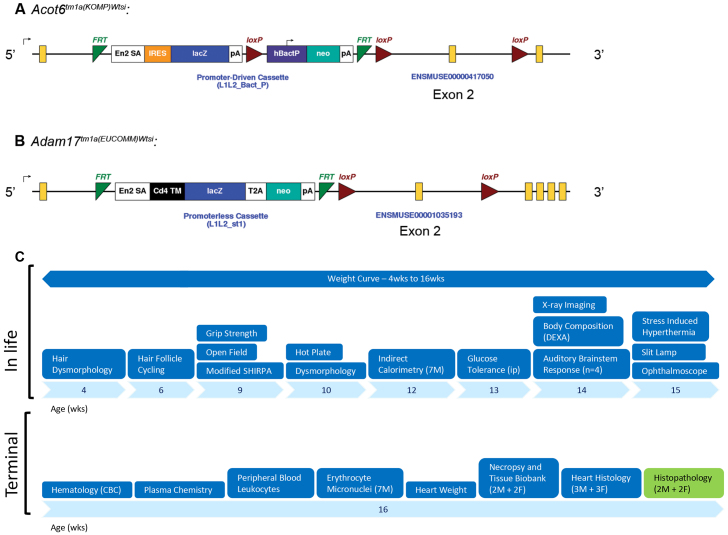
**Examples of the knockout first conditional ready allele designs used and illustration of the clinical phenotyping pipeline.** *Acot6^tm1a(KOMP)Wtsi^* contained a promoter-driven selectable marker (*neo*) (A), whereas *Adam17^tm1a(EUCOMM)Wtsi^* contained a promoterless selectable marker (B) (mouse Ensembl release GRCm38.p1). The alleles were expected to be null alleles, but assessment of the degree of knockdown and the extent of off-target effects on nearby genes was not carried out systematically. (C) The Sanger Institute MGP clinical (blue) and pathology (green) phenotyping pipeline showing tests performed between 4 and 16 weeks of age. Seven male (M) and seven female (F) mutant mice were processed through this pipeline for each allele screened. The pipeline was controlled by processing seven male and seven female wild-type mice per week. CBC, complete blood count.

All the mice used for this study contained a knockout first conditional ready allele, typically designated tm1a(EUCOMM)Wtsi or tm1a(KOMP)Wtsi, and, for brevity, abbreviated hereafter as tm1aWtsi (Fig. 1A,B). The typical workflow of the standard MGP primary phenotyping screen with inclusion of histopathology is displayed in Fig. 1C.

**Fig. 2:**
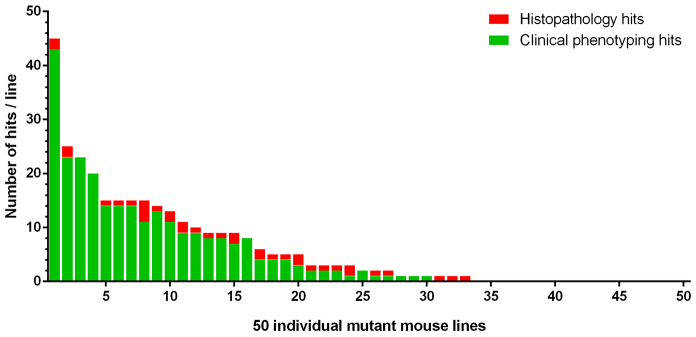
**Distribution of the number of phenotypic abnormalities.** Distribution of the number of phenotypic hits for each of the 50 mutant lines of mice analyzed, split by hits detected during clinical phenotyping (green bars) and histopathology assessment (red bars). No phenotypic abnormalities were detected in 17 of the 50 mutant mouse lines studied. Of the remaining 33 mutant mouse lines, the total number of phenotypic abnormalities ranged from 1 to 45 per line.

Of the 50 mutant mouse lines selected for this pilot, animals homozygous for the targeted alleles of 20 of these lines were viable, fertile, and did not display any detectable phenodeviance in the >280 diverse characteristics assessed by the MGP primary screen. It cannot be ruled out that additional tests, or analysis performed with increased sensitivity, might have uncovered abnormalities manifesting in these mutant mouse lines. The remaining 30 mutant mouse lines presented with at least 1 or up to 42 phenotypic abnormalities. The distribution of the number of phenotypic hits in each line screened including histopathology is indicated in Fig. 2. The heat map for the 50 lines with general categories of tests including histopathology is indicated in supplementary material Table S1. Furthermore, the extended heat map including histopathology hits are included to show every parameter screened for these 50 lines in supplementary material Table S2.

### Incidental and background lesions

Histopathology was performed on a standard panel of 42 tissues and organs per mouse (supplementary material Table S3). Lesions that were attributable to the genetic background of this C57BL/6 substrain (C57BL/6N;C57BL/6-*Tyr^c-Brd^*) and/or other incidental lesions included mild dilation of the lateral ventricles (Brayton, 2006), varying severity of retinal dysplasia (Mattapallil et al., 2012), mild perivascular mononuclear inflammatory cell infiltrates within the salivary glands, renal pelvis and, rarely, within the lungs (Taylor, 2012), mild to moderate neutrophilic and/or eosinophilic gastritis, and rare multinucleated spermatids within the seminiferous tubules in all males (see supplementary material Table S4 for frequency/prevalence of these lesions in wild-type and mutant mice). Diagnostic testing for *Helicobacter* species by fecal PCR and Giemsa staining of histological sections to detect spirochetes within the gastric mucosa were both negative (data not shown). Consistent with a high-fat diet, all control mice and 43 of the 50 mutant lines (86%) in this study had mild to severe hepatic lipidosis as previously reported for the MGP pipeline (Podrini et al., 2013). Hepatic lipidosis was typically characterized by microvesicular vacuolation in periacinar areas and macrovesicular vacuolation in portal and midzonal areas. The severity of hepatic lipidosis varied from mild (affecting up to 30% of hepatocytes) to severe (affecting 90–100% of hepatocytes). Hepatic lipidosis was absent or minimal in seven lines, all with a recorded MGP clinical phenotype (discussed below).

### Histopathology in mice with clinical phenotypes

**Fig. 3:**
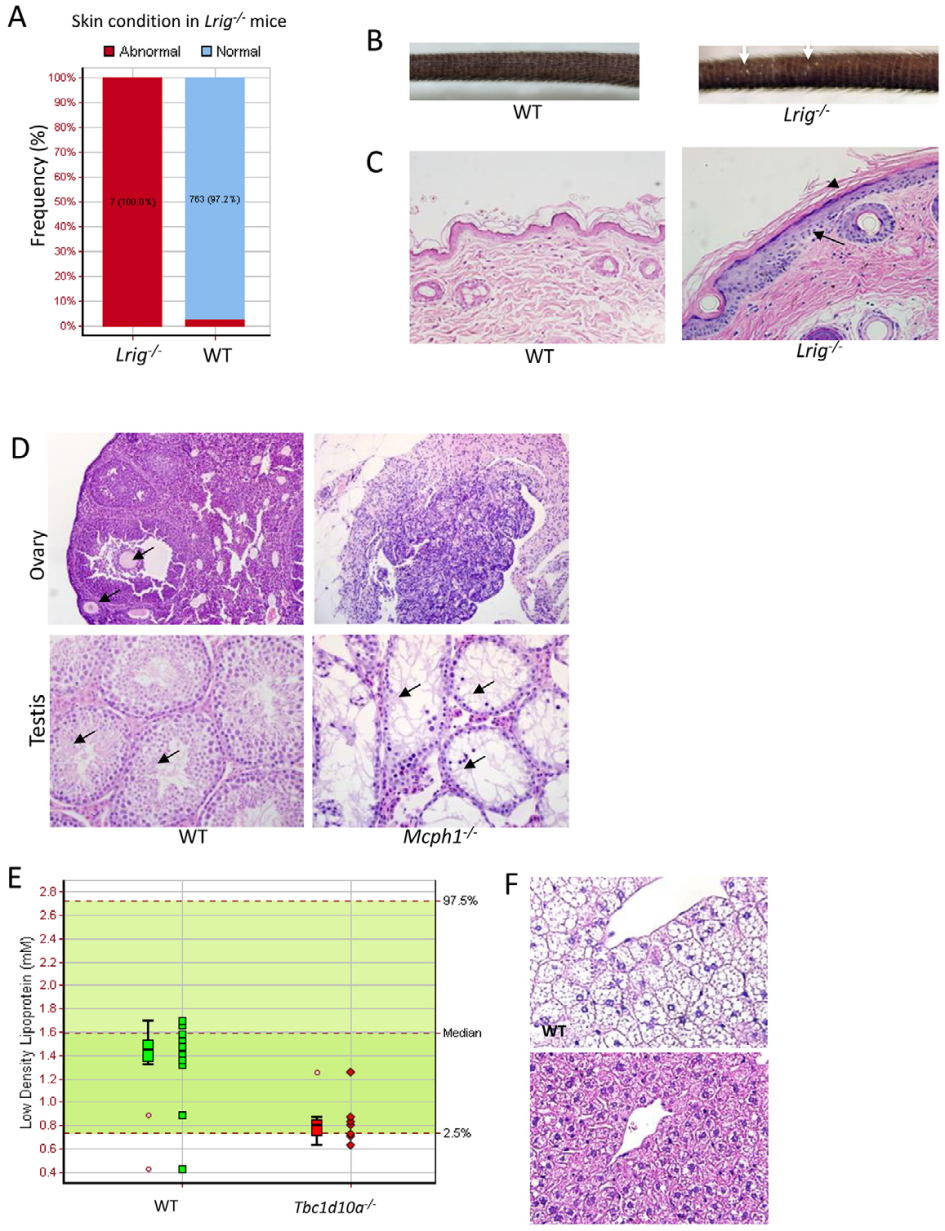
**Histopathology complements clinical phenotypes from the primary screen.** *Lrig*^−/−^ mice were annotated to have abnormal skin (data from male mice is shown) (Fisher’s exact test adjusted *P*-value=2.09e–10). The number and proportion of affected mice in knockout and wild-type baseline controls is presented (A). Abnormal skin with scaly foci (arrows) was observed in *Lrig*^−/−^ mice (B). Histopathology revealed epidermal hyperplasia (arrow) and hyperkeratosis (arrowhead) (C). *Mcph1*^−/−^ mice were annotated as infertile. Ovarian hypoplasia with absence of folliculogenesis was observed in *Mcph1*^−/−^ females (D, top right panel); ovary from a wild-type mouse with growing follicles (arrows) is shown (D, top left panel). Seminiferous tubule vacuolation with lack of germ cells (arrows) was observed in *Mcph1*^−/−^ males (D, bottom right panel); normal testis with abundant developing germ cells (arrows) is shown from a wild-type mouse (D, bottom left panel). *Tbc1d10a*^−/−^ mice were annotated to have decreased circulating low-density lipoprotein (LDL) and high-density lipoprotein (HDL) cholesterol (LDL data from male mice is shown). LDL cholesterol is significantly decreased in knockout mice compared with wild-type mice (E) [adjusted *P*=0.012, male knockout effect estimated: −0.35±0.10 mM (±standard error)]. The wild-type data, collected as local controls (phenotyped in the same week), are shown in the figure as the boxplot, whereas the green band shows the natural variation in the parameter as assessed by the 95% confidence intervals in control mice of that genetic background. Blood fat parameters between WT and knockout mice were compared using a mixed model statistical model. Histopathology revealed absent or minimal hepatic lipidosis in *Tbc1d10a*^−/−^ mice (F, bottom panel). Marked hepatic lipidosis is evident in wild-type mice (F, top panel).

In 19 of 30 lines (63.3%) with MGP clinical phenotypes, histopathology revealed lesions that were associated with at least one documented clinical phenotype (Table 1). In 17 of these lines, histopathology revealed an unequivocal pathology phenotype that potentially explained at least one documented clinical phenotype, and added morphologically and biologically relevant information to the phenotype annotation. For example, histopathology in *Lrig1^tm1a^* mice revealed epidermal and follicular hyperplasia with hyperkeratosis consistent with abnormal skin (scaly skin) observed in clinical phenotyping (Fig. 3A–C). In some lines, multiple pathology phenotypes were observed, consistent with multiple clinical phenotype hits. A case in point is the *Mcph1^tm1a^* line, which showed a range of clinical phenotypes, including male and female infertility, decreased auditory brainstem response, absent pinna reflex, abnormal eye morphology including corneal vascularization, micronuclei, decreased bone mineral content and decreased bone trabeculae (Chen et al., 2013). Histopathology of this line revealed gonadal hypoplasia and absence of gametogenesis in both females and males (consistent with infertility) (Fig. 3D), corneal thickening and hyaloid artery remnant (consistent with abnormal eye morphology), trabecular osteopenia in long bones (consistent with decreased bone trabeculae), and small brain/ microencephaly (consistent with microcephaly) (data not shown). The clinical and pathology phenotypes in this line were broadly consistent with microcephaly, a primary autosomal-recessive human condition associated with mutations of MCPH1 [Online Mendelian Inheritance in Man (OMIM) 251200 (www.ncbi.nlm.nih.gov/omim)] (supplementary material Table S5).

**Table 1:**
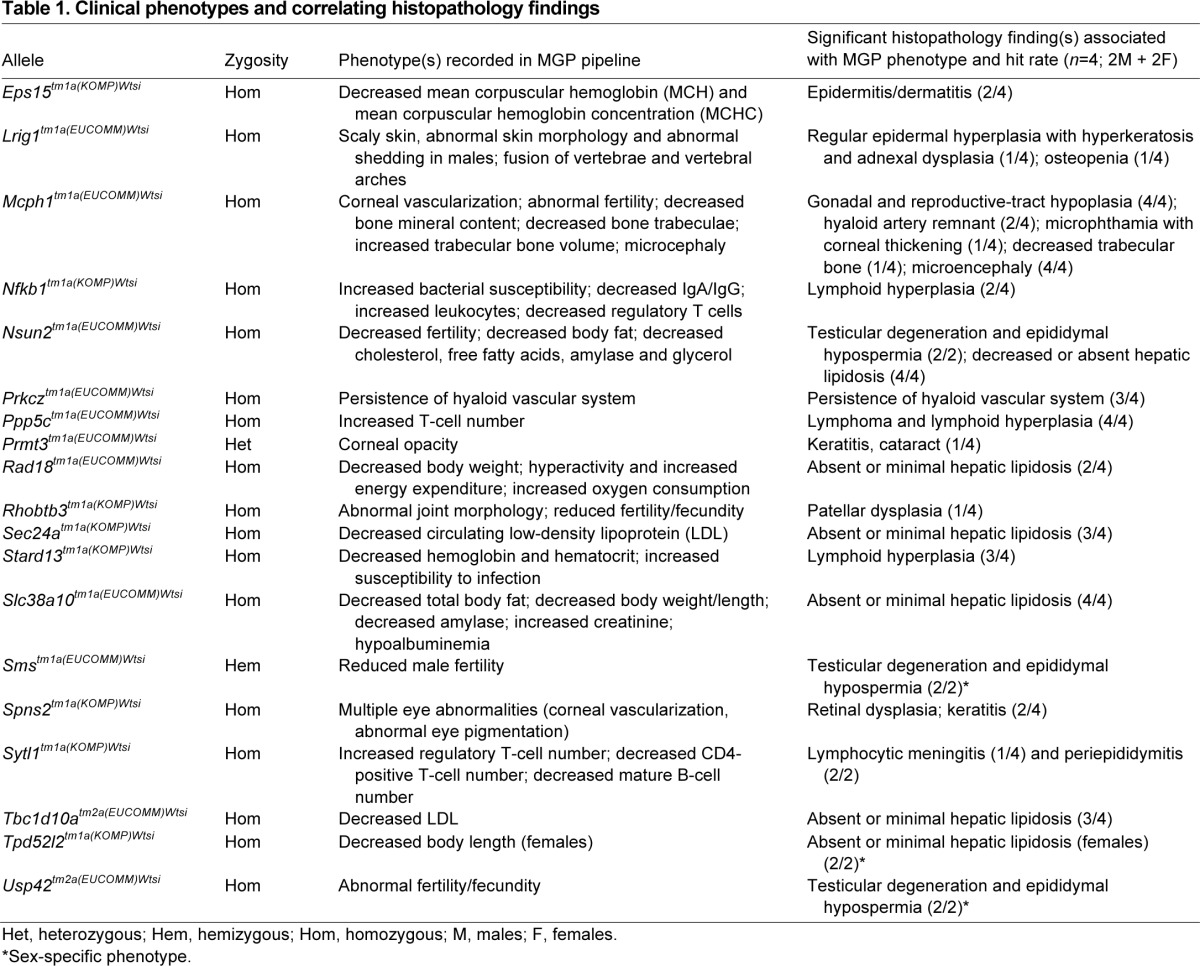
Clinical phenotypes and correlating histopathology findings

Male infertility was one of the most common abnormalities, affecting 4 of 30 lines (13.3%) with a clinical phenotype. Histologically, all of the lines with male infertility (*Ups42^tm1a^*, *Mcph1^tm1a^*, *Sms^tm1a^* and *Nuns2^tm1a^*) had a variable degree of testicular degeneration/atrophy with or without a reduced density or absence of sperm in the lumen of the epididymal duct (aspermia/hypospermia). In addition, two lines (*Socs7^tm1a^* and *Abhd5^tm1a^*) had testicular degeneration, although neither was classified as infertile; histological analysis therefore can add sensitivity to the clinical fertility screen by identifying lines that might be able to generate offspring but have testicular pathology. Recently, the MGP reported an infertility rate of 5.2% in 307 homozygous mutant lines generated from the IKMC targeted ES cell resource (White et al., 2013).

Similarly, a good pathology-clinical phenotype concordance was seen in lines with minimal or absent hepatic lipidosis despite a high-fat diet. In total, seven of the 50 lines (14%) exhibited reduced or absent hepatic lipidosis. In five of these lines (*Slc38a10^tm1a^*, *Sms^tm1a^*, *Rad18^tm1a^*, *Nsun2^tm1a^* and *Tpd52l2^tm1a^*), the minimal/absence of hepatic lipidosis in homozygous/hemizygous mice was associated with overall growth retardation as reflected by reduced body weight and/or length. In two lines (*Sec24a^tm1a^* and *Tbc1d10a^tm2a^*), absence/minimal hepatic lipidosis in homozygous mice was associated with decreased circulating cholesterol and/or low-density lipoprotein in the absence of notable growth retardation. Data from *Tbc1d10a^tm2a^* is shown in Fig. 3E,F. Notably, *Sec24a* and *Slc38a10* are associated with transportation of lipid, protein and/or amino acid, consistent with the observed phenotypes.

Histopathology did not reveal directly correlative lesions in 11 of the 30 lines (36.7%) with clinical phenotypes. Interestingly, nearly all of these 11 lines had clinical phenotypes that were considered challenging to corroborate by histopathology. Examples include abnormal shape or number of vertebral transverse processes as assessed by X-ray imaging (*Efna1^tm1a^*, *Stard5^tm1a^* and *Ppp5c^tm1a^* homozygotes), altered body composition as measured by DEXA (*Bbx^tm1a^* homozygotes), decreased response to stress-induced hyperthermia (*Socs7^tm1a^* homozygotes) and altered bacterial susceptibility based on *Citrobacter rodentium* challenge (*Inpp1^tm1a^* homozygotes). Homozygous animals from two lines, *Mms22l^tm1a^* and *Adam17^tm1a^*, were classified as pre-weaning lethal or subviable, respectively. As a consequence, heterozygotes were processed through the clinical phenotyping pipeline but no abnormalities were noted; hence, histopathology lesions might be subtle or absent in adult heterozygous mice.

Consistent with the young age of the mice (16 weeks), proliferative and neoplastic lesions were rare. Overall, lymphoma was the most common neoplasm, found in a total of 15 mice (two of the 20 wild types and 13 of the 201 mutants); most lymphomas were at an early stage, consistent with the young age of the mice. Other proliferative lesions were extremely rare and included hibernoma (2/221, both *Efna1^tm1a^* homozygotes), bone-marrow myeloid hyperplasia (2/221, both *Ninl^tm1a^* homozygotes), and splenic erythroid and myeloid hyperplasia (2/221, both *Adam17^tm1a^* heterozygotes).

### Histopathology reveals previously unidentified, unpredicted lesions

**Fig. 4:**
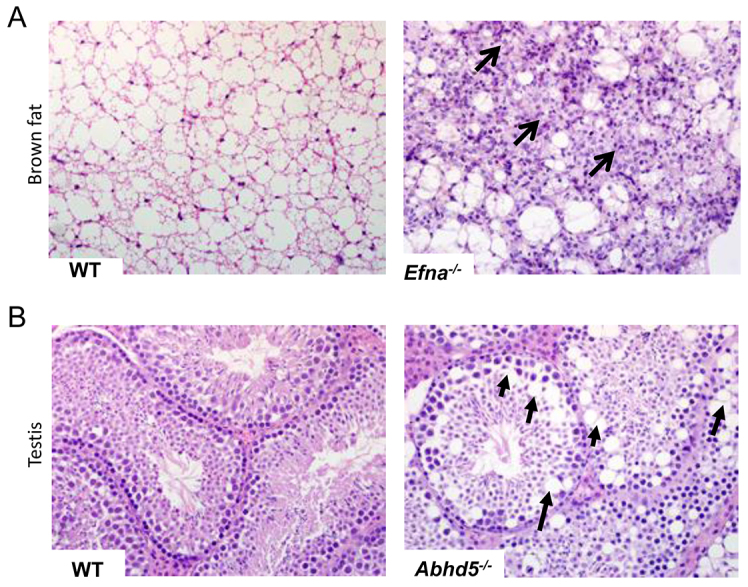
**Histopathology identified novel phenotypes in lines with clinical phenotype annotations from the primary screen.** Benign proliferative lesion of the brown fat (hibernoma) in *Efna*^−/−^ mice (A, right panel, arrows); normal brown fat from a wild-type mouse is shown (A, left panel). Sertoli cell vacuolation in *Abdh5*^−/−^ mice (B, right panel, arrows); normal seminiferous tubules from a wild-type mouse testis is shown (B, left panel). The clinical phenotype annotations from the primary screen for both *Efna*^−/−^ and *Abhd5*^−/−^ lines were skeletal abnormalities.

Seven of the 50 lines (14%) revealed significant histopathology findings that were considered novel or unrelated to the recorded clinical phenotype (Table 2). One example is hibernoma (tumor of brown adipose tissue) in *Efna1*^tm1a^ homozygous mice (Fig. 4A) that were reported to have pelvic and lumbar vertebral abnormalities by X-radiography. Such early neoplastic lesions were unlikely to be detected during the current MGP phenotyping screen. Another line in this category was *Abhd5^tm1a^*; homozygous animals were detected by microcomputed tomography (μCT) and quantitative X-radiography to have increased trabecular bone thickness and decreased number of trabecular bones. No morphological correlate to this bone phenotype was observed by histopathology. However, histopathology did detect a testicular lesion characterized by marked vacuolation of the Sertoli cells in this mutant line (Fig. 4B). Males were fertile despite this testicular abnormality.

**Table 2:**
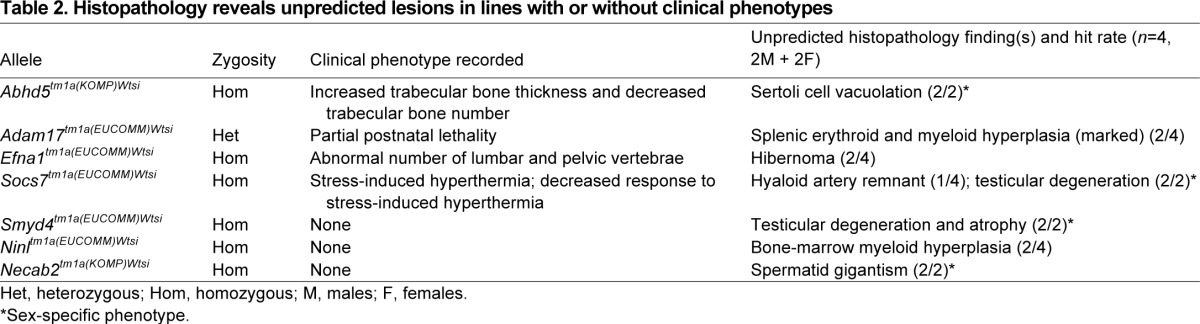
Histopathology reveals unpredicted lesions in lines with or without clinical phenotypes

**Fig. 5:**
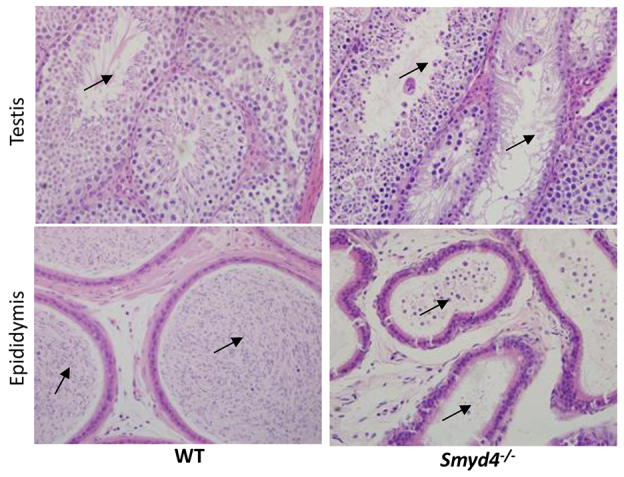
**Histopathology identified novel phenotypes in lines with no recorded clinical phenotype annotations from the primary screen.** Seminiferous tubule degeneration and atrophy (arrows, top right panel) with markedly reduced density or absence of sperm in the epididymal duct (arrows, bottom right panel) in *Smyd4*^−/−^ mice. Normal seminiferous tubules with abundant developing germ cells (arrow, top left panel) and sperm store in the epididymal duct (arrows, bottom left panel) are shown in a wild-type mouse. Infertility or reduced fecundity was not observed in this line.

Other examples of novelty added by histopathology include testicular degeneration and epididymal hypospermia in *Smyd4^tm1a^* homozygotes (2/2) (Fig. 5), spermatid gigantism in *Necab2^tm1a^* homozygotes (2/2) and myeloid-granulocytic hyperplasia in *Ninl^tm1a^* homozygotes (2/4); all were detected in lines that had no observed clinical phenotype (Table 2).

## DISCUSSION

We set out to determine whether the addition of histopathology to a primary screen of targeted knockouts in a large-scale high-throughput pipeline was scientifically valuable, feasible and cost effective; a study that is relevant and timely given the ongoing efforts of the IMPC (Kim et al., 2010; Brown and Moore, 2012).

Our findings revealed that histopathology added correlating morphological data to 19 of the 30 lines (63.3%) with clinical phenotypes. Typically, the clinical phenotyping screen produced multiple phenotype hits on a per line basis compared with histopathology, which typically revealed a single or only a few hits per line. Possible explanations for this discrepancy include some abnormal clinical phenotypes that might not be primary effects; for example, reduced weight could be a consequence of a number of different primary defects. Furthermore, abnormality in a single tissue or organ might manifest as multiple clinical phenotypes, emphasizing the value of histopathology in providing pathophysiological context to clinical phenotyping observations. However, we also consider the fact that many clinical phenotypes might be a consequence of cellular or biochemical abnormalities with subtle or no structural abnormalities.

Histopathology revealed significant findings in seven of the 50 lines (14%) that were not predicted by the clinical phenotyping screen. Four of these seven lines were from lines with clinical phenotype annotations. A case in point was the *Efna1^tm1a^* homozygous line, in which hibernoma (tumor of brown adipose tissue) was found although clinical phenotyping had only identified skeletal abnormalities. Interestingly, *Efna1* plays a role in tumor growth regulation (Sukka-Ganesh et al., 2012). The finding of abnormalities unrelated to clinical phenotypes illustrates that important pathology phenotypes will be missed if histopathology is restricted to perceived target organs or tissues based on clinical phenotyping. The other three lines with unpredicted pathology findings were from lines with no recorded phenotype. Lesions that were not severe enough to cause physiological or behavioral abnormalities were detected by histopathology, including testicular degeneration (*Smyd4^tm1a^* homozygotes) and spermatic gigantism (*Necab2^tm1a^* homozygotes), both of which can compromise fertility with age-dependent severity. Furthermore, marked myeloid hyperplasia in *Ninl^tm1a^* homozygous males in the absence of inflammatory lesions in the examined tissues or a concomitant increase in peripheral blood leukocyte count suggested a myeloproliferative (genetic) disorder (Kogan et al., 2002). These findings underscore the fact that important phenotypes are missed if histopathology in high-throughput screening is limited only to mouse lines with clinical phenotype annotations (Schofield et al., 2012). Similarly, histopathology was the only assay revealing a phenotype in many reports of knockout lines (Schofield et al., 2012; Vogel et al., 2008; Vogel et al., 2013). This observation is particularly important because the subtle/equivocal or absence of a phenotype is common in mutant mice (Doetschman, 1999). Recently, 56 of the 160 lines (35%) screened as homozygotes or hemizygotes by MGP appeared completely normal (White et al., 2013). We consider that these subtle phenotypes could be important and markedly modulated by genetic background and environment (Wood, 2000), or under conditions that were not tested in the current study. Indeed, some of the phenotypes reported here were the result of environmental or pathogen challenges. Examples include three mouse lines with altered bacterial susceptibility and multiple lines with minimal/absent hepatic lipidosis despite high-fat diet challenge. In some of these lines, there is a strong association between pathology and gene annotation and/or specific plasma chemistry, suggesting potentially promising mouse lines with which to explore lipid biology and obesity. These findings underscore the importance of contextual interpretation of histopathology findings not only in the presence but also in the absence of anticipated lesions (in this case hepatic lipidosis) under certain environmental conditions and challenges.

The data set reported here included seven orthologs of known human disease genes (*ABCD1*, *ABHD5*, *AKAP9*, *MCPH1*, *NIPA1*, *SLC22A21* and *SMS*) (supplementary material Table S5). In three of these seven lines (*Mcph1^tm1a^*, *Abhd5^tm1a^* and *Sms^tm1a^*), histopathology revealed lesions that are consistent with the respective human condition. For example, histopathology findings in male and female *Mcph1^tm1a^* homozygous mice included a small but architecturally normal brain (microencephaly), consistent with the recent description of this line (Chen et al., 2013). Mutations in *MCPH1* (microcephalin) have been associated with primary autosomal-recessive microcephaly-1 and premature chromosome condensation syndrome (OMIM 251200) (Woods et al., 2005). In each of these three lines, histopathology revealed additional or novel phenotypes, suggesting additional features that might also occur in affected individuals that have not been identified or associated with the human disease to date. For example, in *Mcph1^tm1a^* mice, besides microcephaly, gonadal hypoplasia was observed in both male and female animals, consistent with infertility, a feature not reported in humans. Homozygous *Abhd5^tm1a^* mice showed testicular lesions characterized by accumulation of large lipid-like vacuoles within the Sertoli cells of the seminiferous tubules (Fig. 4B). This lesion, together with the role of ABHD5 in lipid metabolism, suggests strong genotype-pathology association. In humans, mutation of *ABHD5* is associated with Chanarin-Dorfman syndrome, a rare disease characterized by neutral lipid storage (OMIM 275630). Interestingly, the lipid storage in the mouse line seemed to be restricted to the testis, although liver involvement was difficult to confirm in the presence of diet-induced hepatic lipidosis. Clinical phenotyping of hemizygous *Sms^tm1a^* male mice revealed reduced muscle strength, lean mass and bone mineral density, lumbar lordosis, and growth retardation, recapitulating some features of X-linked Snyder-Robinson syndrome (SRS; caused by mutation in the *SMS* gene) (Cason et al., 2003). Consistent with growth retardation observed in this line, fat deposition in the liver was absent or very minimal in male mice. Males from this line were also infertile with testicular degeneration/atrophy and absence of epididymal sperm storage. This finding is consistent with previous findings in mice with deletion of part of the X chromosome that includes the spermine synthase gene (Wang et al., 2004). Interestingly, infertility has not been recognized in humans with SRS. Discrepancies between the pathology of human diseases and mouse phenotypes caused by similar mutations in orthologous genes have been demonstrated in mouse models of human disease (Vogel et al., 2009). Taken together, findings in lines with mutations in orthologous genes indicate that clinical and pathology phenotyping yield both complementary and unique phenotype annotations for a complete and enriched characterization of mouse models of human disease.

In the current study, histopathology did not provide morphological correlates for all of the clinical phenotypes observed. This could in part be due to experimental design and limitations inherent to histopathology. For example, histopathology is not best suited to corroborate some skeletal dysmorphologies such as anomalies in the number of vertebral bones and ribs. Furthermore, evaluation of a single tissue section might not be sensitive enough to detect subtle morphological variations, biochemical alterations and ultrastructural changes that are associated with immunological, neurological and molecular phenotypes such as abnormal lymphocyte subpopulations, hyperactivity and chromosomal instability, respectively. For example, no morphological basis was found in hemolymphatic tissues to explain the abnormalities in the number of various populations of T cells recorded in *Spns2^tm1a^* homozygous mice (Nijnik et al., 2012). This observation illustrates how specialized clinical phenotyping tests detect phenodeviants that are beyond the scope of routine histopathology. Establishing neurological phenotypes by high-throughput pathology screening is particularly challenging. Notably, histopathology might not be sensitive enough to detect subtle pathologies, especially those associated with variation in cell numbers (Boyce et al., 2010; de Groot et al., 2005) or mild reactive changes such as gliosis (Sofroniew and Vinters, 2010) that require specialized quantitative analyses that are not compatible with large-scale phenotyping. Lack of correlative lesions might also be attributable to tissues not being included in the standardized list of tissues and organs collected. For example, because auricular structures were not routinely collected, we could not rule out the presence of otitis or other causes of conductive hearing loss in some lines with hearing impairment. Similarly, analysis of the brain was limited to a mid-sagittal section; hence, many neuroanatomical and functional areas were not examined for lesions or coronal symmetry. Although consensus on section orientation of the brain is lacking (Hale et al., 2011), coronal sectioning is particularly suited for assessment of brain symmetry, a feature that is affected in many diseases (Bronson, 2001). Recently, the Sanger MGP has adopted coronal sectioning of the brain.

Despite the aforementioned limitations, notable advantages of histopathology in a high-throughput phenotyping pipeline include reduced sources of variation due to experimental design, standardized genetic background, standardized targeting strategy, consistent in-life experience between mice, and standardized tissue collection and processing that allows cross line comparison. Histopathology uniquely allows examination of all organ systems at a given time, allowing a systematic appraisal of the pathophysiological status of the whole animal. Importantly, histopathology is performed on mice that are already used for the clinical phenotyping pipeline; hence, omitting histopathology is a missed opportunity to find correlative and/or unique lesions.

Based on our experience, comprehensive histopathology analysis of a mouse line consisting of two male and two female mutant animals, and generation of an image-enabled dataset and report, takes a pathologist 4–5 hours on average, suggesting a throughput of ten mutant lines per week per pathologist. Additional costs include sample collection, processing and storage. Animal production and husbandry costs are absorbed by the existing primary screen. Therefore, the estimated cost for the histopathology work described here is ≤US$1200 per mutant line (≤US$300 per mutant mouse). This estimate is similar to that reported previously (Schofield et al., 2011). Availability of pathologists with expertise in mutant mouse pathology remains a challenge despite recent efforts to mitigate this shortage (Schofield et al., 2011). The current number of pathologists in large mouse phenotyping centers, supported by development of technologies such as whole-slide scanning and online tools for image sharing and annotation between distributed experts, is believed to make full pathology analysis feasible, at least for the expected caseload from the IMPC project (Schofield et al., 2011). Furthermore, this distributed approach could alleviate the need for expertise in each organ system at each phenotyping center (Schofield et al., 2011).

Furthermore, we recommend full integration of histopathology into a primary phenotyping screen (Fig. 1B) irrespective of the presence or absence of annotations from clinical phenotyping. Consistent with previous recommendations (Schofield et al., 2011) and our experience with the current and other ongoing screens, we suggest wild-type genetic-background-matched control mice that have completed the clinical phenotyping pipeline should be included for every ten mutant lines screened to monitor baseline changes due to genetic drift, infectious diseases or other environmental factors. The number of wild-type mice evaluated can be reduced as pathologists gain experience with the genetic background and conditions used. Because lesions in mutant mice can be subtle, histopathology review is preferably performed with prior knowledge of abnormalities documented during clinical phenotyping. The benefits of this informed approach greatly outweigh the risk of introducing bias that is implicit in an un-blinded experimental design. For example, it primes the pathologist to flag subtle, correlative lesions that could otherwise be overlooked in routine screening, and supports formulation of a comprehensive pathophysiological context, which is crucial to deriving the maximum value from the work and consistent with best practice diagnostic approach. Furthermore, prior knowledge of phenodeviants would allow bespoke processing of non-standard tissues and additional standard tissues to facilitate more in-depth screening and validation, respectively. This approach necessitates coordination between the phenotyping pipeline and pathology screen and is suitable only for low- to medium-throughput workflows.

In summary, we demonstrated the effective integration of histopathology into a high-throughput phenotyping platform, and present its complementary and unique value to clinical phenotyping. The enrichment in hit rate extends the potential for the output of the primary screen, an outcome emphasized and promoted by the IMPC (Brown and Moore, 2012). It also provides crucial morphological data to seed hypothesis-driven secondary and tertiary research in specialized laboratories and ultimately will advance the process of functional annotation of the mammalian genome.

## MATERIALS AND METHODS

### Ethics statement

The care and use of all mice in this study was carried out in accordance with UK Home Office regulations, UK Animals (Scientific Procedures) Act of 1986.

### Animals

Mice were generated by blastocyst injection of targeted ES cells from either the Knockout Mouse Project (KOMP) or the European Conditional Mouse Mutagenesis (EUCOMM) program (Skarnes et al., 2011). All the mice used for this study contained a knockout first conditional ready allele, typically designated tm1a(EUCOMM)Wtsi or tm1a(KOMP)Wtsi, and, for brevity, abbreviated here as tm1aWtsi (Fig. 1A,B). The *tm1a* allele is a targeted trap and, although these alleles are predominantly expected to be null based on previous experience (Testa et al., 2004; White et al., 2013), molecular confirmation of the degree of knockdown (White et al., 2013) and extent of off-target effects on nearby genes (Maguire et al., 2014) is beyond the scope of this study. Owing to the potential issues with this *tm1a* allele, the IMPC, including the MGP, recently changed strategy and now studies the *tm1b* allele, which is derived from the above allele by *loxP*/Cre-mediated excision, and is a *lacZ*-reporter-tagged deletion allele in which the critical exon has been removed. The gene name and full allele symbol for each mutant mouse line included in this study is presented in supplementary material Tables S1 and S2. Mice were maintained on a C57BL/6N;C57BL/6-*Tyr^c-Brd^* genetic background.

### Animal husbandry

Mice were housed in a unit designated specific pathogen free; pathogen load was monitored quarterly using a standard sentinel-based health screening protocol that tested for >30 mouse pathogens (viruses, intestinal protozoa and bacteria), including *Helicobacter* subspecies. Mice were maintained on a 12-hour light:12-hour dark cycle with no twilight period. The ambient temperature was 21±2°C and the humidity was 55±10%. Mice were housed for phenotyping using a stocking density of three to five animals per cage [overall dimensions of caging (L×W×H): 365×207×140 mm, floor area 530 cm^2^] in individually ventilated caging (Tecniplast Seal Safe 1284L) receiving 60 air changes per hour. In addition to Aspen bedding substrate, standard environmental enrichment of two nestlets, a cardboard Fun Tunnel and three wooden chew blocks was provided. Mice were given water and Mouse Breeders Diet (LabDiet 5021-3, IPS, Richmond, USA) *ad libitum* unless otherwise stated.

### Phenotyping pipeline and tests

Mutant mice along with age-, sex- and genetic-background-matched controls were analyzed using the standard Sanger MGP primary phenotyping screen (http://www.sanger.ac.uk/mouseportal/; https://www.mousephenotype.org/; http://en.wikipedia.org/wiki/Category:Genes_mutated_in_mice; and http://www.informatics.jax.org/), which is similar, although not identical to, the standard IMPC pipeline (https://www.mousephenotype.org/impress/pipelines). These pipelines include standard tests used to systematically characterize every line of mice as described recently (White et al., 2013). For most tests, seven male and seven female homozygous animals were assessed. However, for mutations causing lethality or sub-viability, heterozygous animals were screened. Fertility of homozygotes was assessed by homozygous inter-crossing. Mice between 8 and 14 weeks of age were mated for 4 to 6 weeks. Strains that did not produce progeny after 6 weeks were reported as infertile.

At 4 weeks of age, mice were transferred from Mouse Breeders Diet to a high-fat (21.4% fat by crude content; 42% calories provided by fat, 43% by carbohydrate and 15% by protein) dietary challenge (Special Diet Services Western RD 829100, SDS, Witham, UK). We included this high-fat-diet challenge to exacerbate any latent metabolic phenotypes. Dietary composition is not standardized across the IMPC, although typically a breeding or maintenance diet is used by contributing centers, not the dietary challenge used herein. This dietary challenge is no longer used by the MGP.

Body weight was collected at regular intervals and tests were ordered from the least to most invasive [hair dysmorphology (4 weeks), hair follicle cycling (6 weeks), open field, modified SHIRPA and grip strength (9 weeks), hot plate and full dysmorphology (10 weeks), indirect calorimetry (12 weeks, males only), glucose tolerance (13 weeks), auditory brainstem response (*n*=4 only), body composition and X-ray imaging (14 weeks), and stress-induced hyperthermia and eye morphology (slit lamp and ophthalmoscopy) (15 weeks)]. At 16 weeks of age, mice were terminally anaesthetized, blood samples were collected [non-fasted plasma chemistry, complete blood counts, erythrocyte micronuclei (males only) and peripheral blood leukocyte profile], heart weight measured and a necropsy performed with tissue collection. The typical workflow from clinical phenotyping pipelines to histopathology analysis is presented in Fig. 1C.

A second primary pipeline, unique to the MGP, included challenges with two infectious agents, *Citrobacter rodentium* (eight females) and *Salmonella typhimurium* (eight males), with matched controls run simultaneously. In both challenges we looked at colonization of target tissues at 14 and 28 days post-inoculation. Furthermore, serum was collected from the *Salmonella*-challenged animals to measure antigen-specific IgG (and subclass) antibodies.

### Statistical and bioinformatic analysis

A reference range approach was used to assess continuous data, including time course. For categorical data, a Fisher’s exact test was used to identify phenotypic variants. More details of both approaches are described previously (White et al., 2013; Karp et al., 2012). Because both of the above approaches to automatically identify significant calls are known to be conservative and assess each parameter in isolation, they were complemented by a manual assessment made by a biological expert who used knowledge of events on the day, or related variables, or across sexes to highlight variant phenotypes. To assess the auditory brainstem response data, a one-way Kruskal-Wallis ANOVA on Ranks was used where three genotypes were assessed and Mann-Whitney rank sum test where only homozygote and wild-type animals were assessed.

In addition to the above methods, a mixed model was used to analyze continuous data of particular interest to the current histopathology assessment. Details of this approach are described previously (Karp et al., 2012). Data analysis was performed using R (package: nlme version 3.1, package: qvalue version 2.11, and package: car version 2.0–16).

### Histology

At completion of clinical phenotyping, mice (16 weeks of age) were killed and a standard panel of 42 tissues and organs (supplementary material Table S3) were collected and fixed in 10% neutral buffered formalin. The tissues were fixed between 15 and 20 hours, processed overnight (Tek VIP 5, Sakura, Thatcham, UK), embedded in paraffin in 14 multi-tissue blocks, and sectioned at 4 μm for routine hematoxylin and eosin (H&E) staining.

### Histopathology analysis

Histopathology screening was done by veterinary pathologists (H.A.A., S.N. and C.M.) on a total of 50 mouse lines composed of an unbiased selection of lines with (*n*=30) or without (*n*=20) clinical phenotypes detected by the standard MGP primary phenotyping screen. A minimum of two females and two males were screened per mutant line. A total of 20 (ten male and ten female) wild-type control mice were also included to account for incidental and background lesions and to monitor for genetic drift, infectious disease or other environmental factors that might influence interpretation of histopathological changes. Histopathology findings were considered significant if they were not incidental and background lesions routinely documented in the wild-type mice co-housed with the mutant mice. Histopathology data was captured using a Microsoft Access (Microsoft, Seattle, Washington, USA) in-house database using standard adult mouse anatomy (MA) (Hayamizu, et al., 2005) and mouse pathology (MPATH) ontologies (Schofield et al., 2010; Schofield et al., 2013). Images were captured using a microscope-mounted Olympus DP71 digital camera (Olympus Life Science Imaging Systems Inc., Markham, ON, Canada).

### Designation of pathology-clinical phenotype association

Pathology-clinical phenotype association (concordance) was established when any of the significant histopathology findings (lesions) correlated with one or more of the clinical phenotypes. The clinical assays in the MGP phenotyping pipeline were designed to detect a wide range of phenotypes; hence, more than one phenotype annotation was documented for 19 of the 30 lines with clinical phenotype annotations.

During the histopathology evaluation, insignificant and significant findings were evaluated to provide a pathophysiologically plausible explanation for observed phenotypes. All abnormal findings (MGP pipeline observations and histopathology) were integrated to generate a more complete and contextual summary of the overall phenotype of a mutant line.

## Supplementary Material

Supplementary Material
