# Supplementary material for: Histopathology reveals correlative and unique phenotypes in a high-throughput mouse phenotyping screen
Source: Dis Model Mech. 2014 Mar 20;7(5):515–24. doi: 10.1242/dmm.015263 (PMC4007403; doi:10.1242/dmm.015263)
Supplement: Supplementary Material [file supp_7_5_515__index.html]

Histopathology reveals correlative and unique phenotypes in a high-throughput mouse phenotyping screen — Supplementary Material 

# Histopathology reveals correlative and unique phenotypes in a high-throughput mouse phenotyping screen

## DMM015263 Supplementary Material

**Files in this Data Supplement:**

- **Supplementary Material**
