# Supplementary material for: Histopathology reveals correlative and unique phenotypes in a high-throughput mouse phenotyping screen
Source: Dis Model Mech. 2014 Mar 20;7(5):515–24. doi: 10.1242/dmm.015263 (PMC4007403; doi:10.1242/dmm.015263)
Supplement: Supplementary Material [file supp_7.5.515_DMM015263.pdf]

Table S1.

Download Table S1

Table S2.

Download Table S2

Table S3. List of tissues collected for histopathology, embedding, orientation and sectioning.

Download Table S3

Table S4. Incidental and background histopathology findings in wild type and mutant C57BL/6N mice at 16 weeks-of-age maintained on high-fat diet.

Download Table S4

Table S5.

Download Table S5
